# Performance Index and PSII Connectivity Under Drought and Contrasting Light Regimes in the CAM Orchid *Phalaenopsis*

**DOI:** 10.3389/fpls.2019.01012

**Published:** 2019-08-06

**Authors:** Nathalie Ceusters, Roland Valcke, Mario Frans, Johan E. Claes, Wim Van den Ende, Johan Ceusters

**Affiliations:** ^1^Department of Biosystems, Division of Crop Biotechnics, Research Group for Sustainable Crop Production & Protection, KU Leuven, Geel, Belgium; ^2^Molecular and Physical Plant Physiology, UHasselt, Diepenbeek, Belgium; ^3^Department of Microbial and Molecular Systems, Bioengineering Technology TC, KU Leuven, Geel, Belgium; ^4^Laboratory of Molecular Plant Biology, Department of Biology, KU Leuven, Leuven, Belgium; ^5^Centre for Environmental Sciences, Environmental Biology, UHasselt, Diepenbeek, Belgium

**Keywords:** chlorophyll fluorescence, crassulacean acid metabolism, *Phalaenopsis*, performance index, PSII connectivity, specific energy fluxes

## Abstract

Crassulacean acid metabolism (CAM) is a specialized mode of photosynthesis characterized by improved water use efficiency mediated by major nocturnal CO_2_ fixation. Due to its inherent metabolic plasticity CAM represents a successful physiological strategy for plant adaptation to abiotic stress. The present study reports on the impact of drought stress and different light intensities (PPFD 50 and 200 μmol m^–2^ s^–1^) on the photosynthetic performance of the obligate CAM orchid *Phalaenopsis* “Edessa” by integrating diel gas exchange patterns with assessments of the light reactions by analyzing fast chlorophyll *a* fluorescence induction. Parameters such as PI_abs_ (performance index), different energy fluxes per active reaction centre (RC) reflecting the electron flow from photosystem II to photosystem I and the energetic communication between PSII complexes defined as connectivity were considered for the first time in a CAM plant. A higher PS II connectivity for plants grown under low light (*p* ∼ 0.51) compared to plants grown under high light (*p* ∼ 0.31) brought about similar specific energy fluxes of light absorbance, dissipation and processing through the electron transport chain, irrespective of the light treatment. With a 25% higher maximum quantum yield and comparable biomass formation, low light grown plants indeed proved to process light energy more efficiently compared to high light grown plants. The performance index was identified as a very reliable and sensitive parameter to indicate the onset and progress of drought stress. Under restricted CO_2_ availability (due to closed stomata) leaves showed higher energy dissipation and partial inactivation of PSII reaction centres to reduce the energy input to the electron transport chain and as such aid in avoiding overexcitation and photodamage. Especially during CAM idling there is a discrepancy between continuous input of light energy but severely reduced availability of both water and CO_2_, which represents the ultimate electron acceptor. Taken together, our results show a unique flexibility of CAM plants to optimize the light reactions under different environmental conditions in a dual way by either attenuating or increasing energy flux.

## Introduction

Plants thrive in a variety of environments, each associated with certain characteristics and limitations (e.g., rainforest, desert, and arctic conditions). Due to their sessile nature they need to be extremely adaptable to their continuously changing environment. Crassulacean acid metabolism (CAM), which is characterized by an optimized water use efficiency by taking up CO_2_ predominantly at night is an important physiological strategy for plant adaptation (Cushman and Borland, 2002; Borland et al., 2011; Ceusters et al., 2017). Traditionally, diel CAM has been defined within a four-phase framework to describe the photosynthetic performance (Osmond, 1978): (i) phase I – open stomata in the dark and external CO_2_ fixation via phospho*enol*pyruvate carboxylase (PEPC) into C_4_ acids (mostly malic acid); (ii) phase 2 – open stomata at the start of the light period and external CO_2_ fixation by combination of PEPC and ribulose-1,5-bisphosphate carboxylase-oxygenase (RubisCO); (iii) phase 3 – closed stomata in the middle of the day whilst malic acid is decarboxylated [catalyzed by NAD(P)-malic enzyme (ME) or phospho*enol*pyruvate carboxykinase] and refixation of CO_2_ by RubisCO; and (iv) phase 4 – stomata open toward the end of the day and external CO_2_ is fixed via RubisCO and/or PEPC. An ubiquitous feature of CAM is the integration of circadian and metabolite control over nocturnal C_4_ and daytime C_3_ carboxylation processes, hereby providing plasticity for optimizing carbon gain and water use by extending or curtailing the period of net CO_2_ uptake over any 24-h period (Borland et al., 1999; Ceusters et al., 2009a, 2010; Haydon et al., 2011; Ceusters et al., 2014; Yang et al., 2015). When experiencing severe drought stress CAM plants enter the stage of CAM-idling, showing no net CO_2_ uptake and recycling respiratory CO_2_ behind closed stomates during the complete diel cycle to minimize any further water loss (Ting, 1985; Ceusters et al., 2009a,b).

**FIGURE 1:**
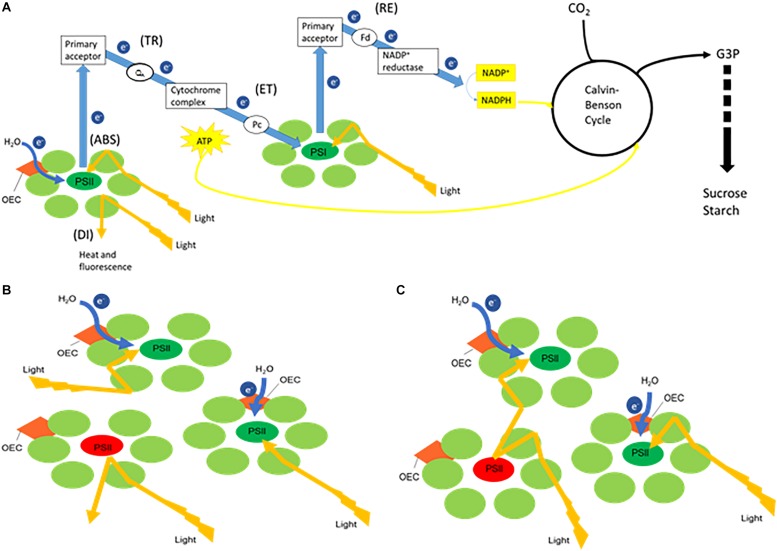
Simplified schemes representing **(A)** the electron transport chain creating ATP and NADH, linked with carbon fixation where the measured energy fluxes are indicated between brackets. When receiving light, part of the flux of photons absorbed by photosystem II (PSII) (ABS) will be utilized to drive the electron transport chain. Trapping flux (TR) indicates the initial electron flow where plastoquinone A (Q_A_) will be reduced. The electrons will further proceed to cytochrome complex followed by further transport mediated by plastocyanin (Pc) to photosystem I (PSI) (ET). Part of the original excitation energy is not utilized in the photochemical reactions and is dissipated, mainly as heat (DI). The splitting of water by the oxygen-evolving complex (OEC) releases electrons (e^–^) to feed the electron transport chain. Finally in PSI the reduction of the end electron acceptors (RE) via ferredoxin (Fd) mediates the production of NADPH by NADP^+^ reductase activity. ATP (created by ATP synthase fueled by a proton gradient resulting from the electron transport chain) and NADPH will provide energy and reducing power for the synthesis of glyceraldehyde 3-phosphate (G3P) during the Calvin-Benson cycle, which will lead to the synthesis of sucrose and starch. Panels **(B,C)** both represent a few PSII units with one closed reaction centre (red) and two open reaction centres (green). **(B)** Without connectivity between the three PSII units, excitation energy that encounters a closed reaction centre will be dissipated. **(C)** Taking connectivity into account, excitation energy from a closed RC will be transferred to an open (active) PSII RC.

Plant photosynthesis is generally characterized by two linked key processes, i.e., (1) light energy capture and use in the electron transport chains to provide energy and reducing power and (2) CO_2_ sequestration into triose phosphates (Figure 1). Non-destructive methods like chlorophyll fluorescence and leaf gas exchange analyses can easily be applied to investigate photosynthetic performance. Chlorophyll fluorescence analyses allow to obtain detailed information about the primary light mediated reactions of photosynthesis. Basically, light energy absorbed by the sun can be dissipated by either photochemical (i.e., photosynthesis) or non-photochemical processes (i.e., heat or fluorescence) (Maxwell and Johnson, 2000). As these processes occur in close competition, abiotic stress factors such as deviations of the optimal temperature, light and water availability can easily cause imbalances between the absorption of light energy by chlorophyll and the subsequent use of energy in CO_2_ fixation (Figure 1; Cornic and Massacci, 1996; Falk et al., 1996). Evaluation of the response of the photosynthetic apparatus to different conditions has generally been performed by analyzing parameters describing the maximum quantum efficiency of photosystem II (PSII) photochemistry (F_v_/F_m_), the effective quantum yield of PSII photochemistry (*Φ*_PSII_), electron transport rate (ETR), photochemical quenching (qP) and non-photochemical quenching (qN). In addition different authors suggested that (1) the performance index (PI_abs_) parameter, which quantifies the overall functionality of the electron flow through PSII, might be a sensitive parameter of plant homeostasis; and (2) that it is also interesting to evaluate specific energy fluxes per reaction centre (RC) i.e., absorption (ABS/RC), trapping (Tr_0_/RC), electron transport (Et_0_/RC), reduction of end electron acceptors (Re_0_/RC) and dissipation (Di_0_/RC) to obtain information about the response of the photosynthetic apparatus under different environmental conditions (Krüger et al., 1997; Rapacz, 2007; Zivcak et al., 2008, 2014a,b; Campos et al., 2014; Chondrogiannis and Grammatikopoulos, 2016). The specific energy fluxes measured per active PSII reaction centre are all interconnected and can be considered as expressing the behavior of the PSII system when all RCs are open (i.e., at the onset of excitation). In healthy plants (Figure 1A), part of the flux of absorbed photons (ABS) will be channeled as trapping flux to the RC (TR) and another part of this excitation energy is dissipated, mainly as heat (DI). An electron transport will be created within the RCs (ET) and further to PSI (RE), ultimately leading to CO_2_ fixation (Strasser et al., 2000). Another intriguingly topic is the possibility of energetic communication (also known as “grouping” or “connectivity” and “overall grouping probability”) between different PSII complexes (Stirbet, 2013), allowing the transfer of excitation energy from a closed PSII RC to an open (active) PSII RC (Figures 1B,C). Zivcak et al. (2014a) demonstrated that the omission of connectivity can lead to misinterpretation of the JIP-test results in mature barley plants. The JIP test, which stands for the major inflection points in the fluorescence induction curve, is based on the theory of energy fluxes in the photosynthetic apparatus (Strasser et al., 2000; Stirbet and Govindjee, 2011). The concept of PSII connectivity is still under discussion in scientific literature and has not yet been taken into account in studies with CAM plants which are exceptionally well adapted to environmental stresses.

In CAM ecophysiology, chlorophyll fluorescence has already been explored to some extent regarding adaptation and survival strategy in response to variations in the natural environment. Measurements of chlorophyll fluorescence quenching confirmed the existence of diverse photoprotective mechanisms in CAM involving non-radiative excess energy dissipation and xanthophyll cycling (Winter et al., 1990; Adams and Demmig-Adams, 1996; Barker and Adams, 1997; Masrahi et al., 2015). Changes in the electron transport rates in CAM, related to CO_2_ reduction rates in chloroplasts, were shown to result primarily in alterations in the rates of non-radiative energy dissipation so that consequently the reduction state of the primary electron acceptor of PSII was maintained at relatively low and constant levels (Winter and Demmig, 1987). The extra requirements of ATP for CAM plants are reflected by high values of total qN, which can be twice as much as compared to C3 (Keiller et al., 1994; Heber et al., 1996; Skillman and Winter, 1997). Chlorophyll fluorescence measurements also revealed the negative impact of abiotic stress, related to water and light availability, on the photosynthetic capacity of different CAM plants by significantly reducing photosynthetic electron transport rate and the overall effective quantum yield of PSII photochemistry, despite an increased capacity in dissipating excess energy (Osmond, 1982; de Mattos et al., 1999; Pollet et al., 2009; Arias-Moreno et al., 2017; Zheng et al., 2019).

The purpose of the present work was to gain more insight in the functioning of the photosynthetic apparatus in the CAM orchid *Phalaenopsis* “Edessa” under different light conditions and under drought stress, by evaluating performance indices (PI_abs_) and specific energy fluxes as described above. Therefore a detailed study was performed integrating diel gas exchange patterns with analyses of fast chlorophyll *a* fluorescence induction. Under both environmental conditions, specific fluxes were calculated without and with connectivity to investigate the importance of energetic communication between PSII complexes in the CAM orchid *Phalaenopsis* “Edessa.”

## Materials and Methods

### Plant Material and Experimental Setup

*Phalaenopsis* “Edessa” is an obligate CAM plant and belongs to the family of the Orchidaceae. Vegetative plants of 16 weeks old were cultivated in a growth room with a day/night temperature of 28°C, a relative humidity of 75% and a 12-h photoperiod with photosynthetic photon flux density (PPFD) of 100 μmol m^–2^ s^–1^ at leaf level. Watering was performed twice a week; one time with a nutrient solution Peters 20N-8.7P-16.6K of 1 mS cm^–1^ and once with water. Following a 2 months acclimation to these conditions, plants were divided into two groups to investigate the possible effect of light intensity on the photosynthetic performance: (1) PPFD of 50 μmol m^–2^ s^–1^ (low light: LL) and (2) PPFD of 200 μmol m^–2^ s^–1^ (high light: HL). As total diel CO_2_ uptake for *Phalaenopsis* has earlier been noticed to saturate around 200 μmol m^–2^ s^–1^, this light intensity is further considered as high light treatment for *Phalaenopsis* “Edessa” (Guo et al., 2012). To determine chlorophyll content and titratable acidity samples were taken from the upper one-third of young fully expanded leaves (*n* = 5 plants) at week 0, 4, 6, and 10. In addition a drought experiment was performed with other plants kept at 100 μmol m^–2^ s^–1^, which had also been divided into two groups: (1) control group which continued to receive watering twice a week and (2) a treatment group which was attributed to drought stress by withholding water. Samples were taken from the upper one-third of young fully expanded leaves (*n* = 5 plants) to determine chlorophyll content and titratable acidity at week 0, 4, and 6. Leaf discs (*n* = 5 plants) were punched out just beneath the sample position to determine relative water content (RWC). At week six, the oldest and most wilted leaves of the treatment group were also included to analyze chlorophyll *a* fluorescence.

### Gas Exchange Measurements and Titratable Acidity

For both light and drought experiments, gas exchange was measured on the youngest fully expanded leaves after 10 and 6 weeks, respectively, using a LCi Portable Photosynthesis System (ADC BioScientific Ltd., United Kingdom). The top part of the leaf was enclosed in a broad leaf chamber (6.25 cm^2^) and the incoming air was passed through a 20-l bottle to buffer short-term fluctuations in the CO_2_ concentration. Gas exchange data were collected over a 24-h period with measurements obtained at 15-min intervals (*n* = 3 plants). By integrating specific areas under the CO_2_ exchange curves, net gas exchange was calculated per phase as well as total net gas exchange during the 24-h time course. Nocturnal CO_2_ uptake was also assessed by analyzing the nocturnal increase in titratable acidity (ΔH^+^) by titration of methanol extracts against 0.005 M NaOH with phenolphthalein as indicator.

### Relative Water Content Determination

Relative water content was determined by sampling the youngest fully expanded leaves (*n* = 5 plants) and calculated with the formula: (fresh mass – dry mass)/(turgid mass – dry mass) according to Ceusters et al. (2008). Leaf parts (95 mm^2^) were floated in plastic tubes filled with demineralized water for 6 h in darkness at 4°C to determine turgid mass.

### Determination of Chlorophyll Content and Plant Growth

For the calculation of leaf chlorophyll contents, plant pigments were extracted by immersing leaf material in N,N-dimethylformamide (DMFA) at room temperature for 72 h in darkness (*n* = 5 plants). The supernatant was used to determine absorbance at 647 nm (A_647_) and 664 nm (A_664_) (Genesys 10S UV-VIS, Thermo Fisher Scientific, United States). These data were used to calculate the content of chlorophyll *a*, chlorophyll *b* and total chlorophyll by means of the empirical formulas: C_a_ = 11.65 A_664_ – 2.69 A_647_; C_b_ = 20.81 A_647_ – 4.53 A_664_; C = C_a_ + C_b_ (Moran and Porath, 1980; Porra et al., 1989; Wellburn, 1994).

Differences in plant growth, after growing for 10 weeks under different light conditions, were evaluated on basis of differences in above ground fresh and dry mass (g). Above ground plant material was separated from the roots, weighed and dried at 70°C to a constant mass after 8 days (*n* = 5 plants).

### Chlorophyll *a* Fluorescence

Chlorophyll *a* fluorescence measurements were carried out by means of a Handy PEA fluorometer (Plant Efficiency Analyser, *Hansatech*, King’s Lynn, United Kingdom) and were taken on the adaxial side, always on the left side of the main vein, of young fully developed leaves (*n* = 15 plants). Measurements were performed using a saturating pulse of 3 000 μmol m^–2^ s^–1^, pulse duration of 1 s, and fixed gain (1.2×). Induction curve analysis (Handy PEA software V1.10) allowed to evaluate the effectiveness of fluorescence saturation during measurements. Before carrying out measurements, leaves were always allowed to dark adapt for 20 min using light-withholding clips (*Hansatech*). The light level of the saturating pulse and the minimal dark period had experimentally been determined for *Phalaenopsis* “Edessa” beforehand in order to obtain true values for the chlorophyll *a* fluorescence parameters. During the light experiment chlorophyll measurements were taken at 08.00 h (Phase II) on week 0, week 4, week 6, and week 10. All chlorophyll fluorescence data was assembled at 08.00 h because additional measurements at 12.00 h (Phase III) revealed no significant differences for these parameters in the different CAM phases (Supplementary Table 1). To study the progression of drought stress, chlorophyll fluorescence measurements were also taken at 08.00 h on week 0, week 4, and week 6.

The measured fast chlorophyll fluorescence induction curves (F_0_ to F_m_) were analyzed by the JIP test, which is based on the theory of energy fluxes in the photosynthetic apparatus (Strasser et al., 2000; Stirbet and Govindjee, 2011). When receiving light, part of the flux of photons absorbed by PSII antenna pigments (ABS) is dissipated (DI), mainly as heat, and another part is converted to redox energy by reducing the electron acceptor Q_A_ (TR). This electron acceptor is then reoxidized creating an electron transport (ET) until the reduction of the end electron acceptors at the PSI electron acceptor side (RE) (Figure 1A). This stepwise flow of energy through PSII can also be expressed per RC, defined as following specific energy fluxes: ABS/RC, Tr_0_/RC, Di_0_/RC, Et_0_/RC, and Re_0_/RC. All these specific fluxes refer to time zero, i.e., the onset of fluorescence induction. In a logarithmic time scale, fast chlorophyll fluorescence induction curves have a typical shape which shows the steps O, J, I, P (Strasser and Strasser, 1995; Srivastava et al., 1999; Strasser et al., 2000, 2004, 2010), making it possible to collect following cardinal points: maximal fluorescence intensity (F_m_, when all RCs are closed), minimum fluorescence intensity (F_0_, when all RCs are open), fluorescence intensity at 2 and 30 ms (F_J_ and F_I_, respectively) and at 50 and 300 μs (F_50 μs_ and F_300 μs_). These primary data were used to calculate chlorophyll fluorescence parameters describing maximum quantum efficiency of PSII (F_v_/F_m_), performance index (PI_abs_) and the afore mentioned specific energy fluxes (Chondrogiannis and Grammatikopoulos, 2016). Definitions and equations of the measured and calculated JIP parameters are described in Table 1.

**TABLE 1:** Definitions and equations of measured and calculated parameters derived from fast fluorescence kinetics.

**Parameter**	**Name and basic physiological interpretation**
**Basic JIP-test parameters**	
V_t_ = (F_t_ – F_50 μs_)/(F_m_ – F_50 μs_)	Relative variable fluorescence at time t (V_J_, V_I_ at 2, 30 ms)
M_0_ = dV/dt_0_	Approximate value of the initial slope of relative variable fluorescence curve Vt (for F_0_ = F_50 μs_)
**Specific energy fluxes (per active PSII reaction centre)**	
ABS/RC = (M_0_/V_J_) × (1/φP0)	Absorbed photon flux per RC
Tr_0_/RC = M_0_/V_J_	Trapped excitation flux (leading to Q_A_ reduction) of absorbed photons per RC
Et_0_/RC = (M_0_/V_J_) × (1 – V_J_)	Electron transport flux (from reduced Q_A_ to Q_B_) per RC
Re_0_/RC = (M_0_/V_J_) × (1 – V_I_)	Electron flux reducing end electron acceptors at the PSI acceptor side, per RC
Di_0_/RC = (ABS/RC – Tr_0_/RC)	Dissipated energy flux per RC
**Performance index**	
PI_ABS_ = RC/ABS × [φP_0_/(1 – φP_0_)] × [ΨE_0_/(1 – ΨE_0_)]	Performance index for energy conservation from photons absorbed by PSII antenna, to the reduction of Q_B_
**Quantum yields and probabilities**	
φP_0_ = F_V_/Fm	Maximum quantum efficiency of primary PSII photochemistry; maximum efficiency at which light absorbed by PSII is used for reduction of Q_A_
ΨE_0_ = 1 – V_J_	Probability with which a PSII trapped electron is transferred from reduced Q_A_ to Q_B_
**Connectivity among PSII units**	
*W*_E_ = 1 – [(F_J_ – F_300 μs_)/(F_J_ – F_50 μs_)]^1/5^	Model-derived value of relative variable fluorescence at 100 μs calculated for unconnected PSII units
*W* = (F_100 μs_ – F_50 μs_)/(F_J_ – F_50 μs_)	Relative variable fluorescence at 100 μs
*C* = (W_E_ – W)/[V_J_ × *W* × (1 – *W*_E_)]	Curvature constant of intial phase of the O-J curve
*p*_2G_ = C × [F_50 μs_/(F_J_ – F_50 μs_)]	Overall grouping probability between PSII units when they are all open
*p* = [*p*_2G_ × (F_m_/F_50 μs_ – 1)]/[1 + *p*_2G_ × (F_m_/F_50 μs_ – 1)]	Connectivity parameter
ω = *p* × [(F_m_ – F_50 μs_)/F_m_]	Probability of the connectivity among PSII units when they are all closed

*Based on information presented by Strasser et al.(1995, 2000, 2004, 2010), Strasser and Stirbet (2001), Stirbet and Govindjee (2011), Stirbet (2013).*

Since fluorescence induction data may be affected by the existence of PSII excitonic connectivity (Figures 1B,C), i.e., transfer of excitation energy from a closed PSII RC to an open (active) PSII RC (Stirbet, 2013), it was interesting to take this process into account. Connectivity parameters were calculated to allow comparison between results without and with taking connectivity into account (Table 1). Based on Zivcak et al. (2014a) the curvature constant (C) of the initial phase of the O-J curve (from 0.05 to 2 ms) was used to correct the values of the specific fluxes for connectivity, i.e., multiplying the specific flux values by 1 + C (Table 1).

### Statistical Analyses

Where appropriate, data were analyzed using the statistical software package IBM SPSS Statistics V23. Before carrying out statistical tests, normality of the data was checked by means of the Kolmogorov-Smirnoff statistic (*p* > 0.01). Since our goal was to interpret the chlorophyll fluorescence data in function of the specific treatments (control/drought and different light intensities) and exclude non-treatment effects (plant age, time effects, …), results were always compared between treatments from the same age by independent sample *t*-test or Tukey’s Studentized range test, both at the 1% probability level. All replicates considered in our study were independent biological replicates originating from different plants.

## Results

### Light

#### Gas Exchange Measurements and Titratable Acidity

**FIGURE 2:**
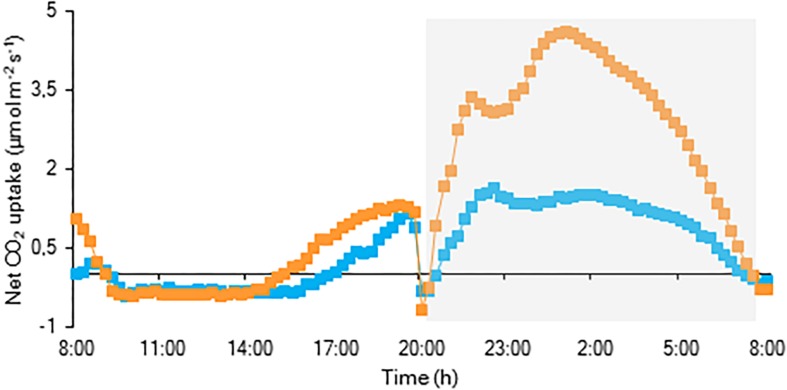
Net 24 h CO_2_ uptake (μmol m^–2^ s^–1^) for young fully expanded leaves of *Phalaenopsis* “Edessa” 10 weeks after growing under different light conditions i.e., PPFD of 50 μmol m^–2^ s^–1^ (blue) and PPFD of 200 μmol m^–2^ s^–1^ (orange). The dark period is indicated in gray. Gas exchange curves are representative of three replicate runs with SE < 15%.

**FIGURE 3:**
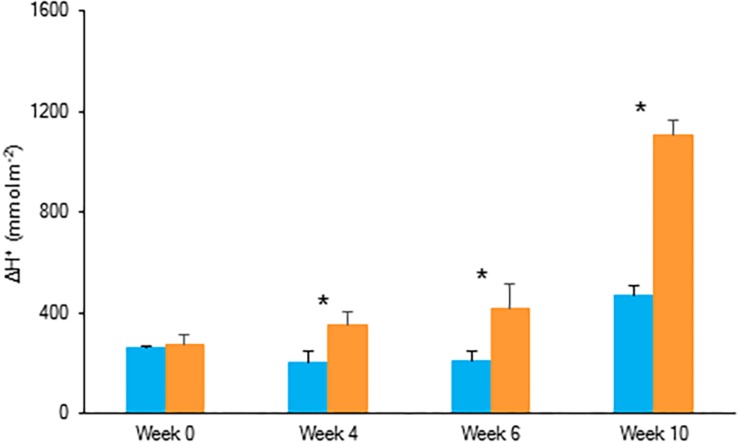
Nocturnal increase (between 20.00 h and 08.00 h) in titratable acidity (mmol H^+^ m^–2^) in young fully expanded leaves of *Phalaenopsis* “Edessa” at 0, 4, 6, and 10 weeks after growing under different light conditions, i.e., PPFD of 50 μmol m^–2^ s^–1^ (blue) and PPFD of 200 μmol m^–2^ s^–1^ (orange). Data are means ± SD (*n* = 5 plants). Asteriks indicate significant differences per week between different light conditions at *P* < 0.01 according to the independent sample *t*-test.

For both investigated PPFD conditions (i.e., LL: 50 μmol m^–2^ s^–1^ and HL: 200 μmol m^–2^ s^–1^) a traditional diel CAM pattern was observed (Figure 2). A complete 24 h CAM cycle occurred with nocturnal CO_2_ fixation (Phase I) and net CO_2_ loss during the day (Phase III), accompanied by two intermediate phases (Phases II and IV). During Phase I, extending from 20.00 h to 8.00 h, nocturnal CO_2_ uptake of LL grown plants was only approximately one third that of plants grown under HL. This difference was also reflected in the nocturnal increase of titratable acidity which was already significantly higher for HL plants after 4 weeks (Figure 3). In addition, during Phase II, CO_2_ uptake was lowered by 80% when grown under LL after 10 weeks. Phase IV was also delayed with 2 h under LL, resulting in only 44% CO_2_ uptake in comparison to plants grown under HL. As a consequence, total daily CO_2_ gain of HL plants was three times higher compared to LL plants (129 ± 20 mmol m^–2^ and 41 ± 19 mmol m^–2^ respectively; Figure 2).

#### Chlorophyll *a* Fluorescence Parameters

**FIGURE 4:**
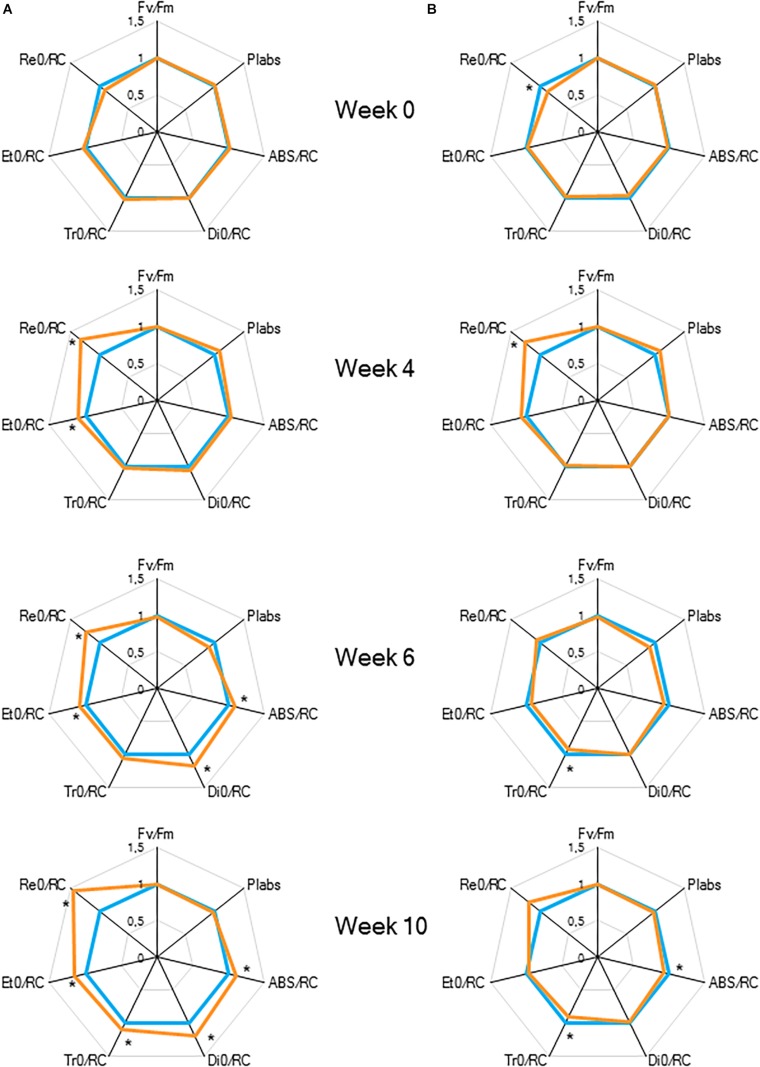
“Spider plots” of selected chlorophyll *a* fluorescence transients parameters measured at 08.00 h in young fully expanded leaves of *Phalaenopsis* “Edessa” at 0, 4, 6, and 10 weeks after growing under different light conditions, i.e., PPFD of 50 μmol m^–2^ s^–1^ (blue) and PPFD of 200 μmol m^–2^ s^–1^ (orange). Parameters are calculated without connectivity **(A)** and corrected for connectivity between PSII units **(B)**. All values are shown as percent of PPFD of 50 μmol m^–2^ s^–1^ (*n* = 15 plants). Asteriks indicate significant differences between different light conditions at *P* < 0.01 according to the independent sample *t*-test.

Comparing the two light conditions, no significant differences were observed for the maximum quantum efficiency of PSII photochemistry (F_v_/F_m_) and for the performance index for absorption (PI_abs_) (Figure 4 and Table 1). However, regarding the specific energy fluxes (per active RC) (Figure 1A and Table 1), significantly higher values were obtained for plants grown under HL (Figure 4A). After 6 weeks, a significant increase was noticed for photon absorption (ABS/RC) for plants grown under HL, concomitantly with a slight increase in the activity of energy dissipation (Di_0_/RC). A significant rise was also calculated for the electron trapping efficiency (Tr_0_/RC) after 10 weeks grown under HL. For the activity of electron transport within the reaction centre (Et_0_/RC) and the flow of electrons further than PSII (Re_0_/RC) significant differences were observed from week 4. Also for these parameters, the highest values were calculated for plants grown under HL.

**FIGURE 5:**
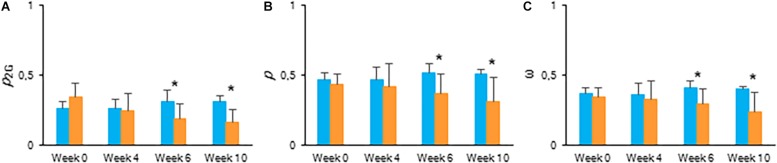
Calculated connectivity parameters derived from fast fluorescence kinetics measured at 08.00 h in young fully expanded leaves of *Phalaenopsis* “Edessa” at 0, 4, 6, and 10 weeks after growing under different light conditions, i.e., PPFD of 50 μmol m^–2^ s^–1^ (blue) and PPFD of 200 μmol m^–2^ s^–1^ (orange). **(A)** Overall grouping probability of PSII units when they are all open. **(B)** Connectivity parameter. **(C)** Probability of connectivity among PSII units when they are all closed (Strasser and Stirbet, 2001; Stirbet and Govindjee, 2011; Stirbet, 2013). Data are means ± SD (*n* = 15 plants). Asteriks indicate significant differences between different light conditions at *P* < 0.01 according to the independent sample *t*-test.

To estimate the connectivity among PSII units, the first part of fast chlorophyll fluorescence induction curve was used (from 0.05 to 2 ms) as described above (Strasser and Stirbet, 2001; Joliot and Joliot, 2003; Stirbet, 2013). From week 6, calculated values of parameters associated with connectivity (Table 1), i.e., overall grouping probability of PSII units – *p*_2G_, connectivity parameter – *p*, probability of connectivity among PSII units – ω (as defined by Strasser and Stirbet, 2001), were ∼1.5 times higher in plants grown under LL compared to those grown under HL (Figure 5). After correction for connectivity, i.e., multiplying the specific flux values by 1 + C (Zivcak et al., 2014a), all values obtained under LL became higher (Figure 4B) and eliminated the significant differences in the activity of energy dissipation (Di_0_/RC) and electron transport (Et0/RC) originating from not considering connectivity. At week 6 photon absorption (ABS/RC) was equally for both light conditions and trapping efficiency was significantly increased in LL plants when taking connectivity into account.

#### Chlorophyll Content and Plant Growth

**FIGURE 6:**
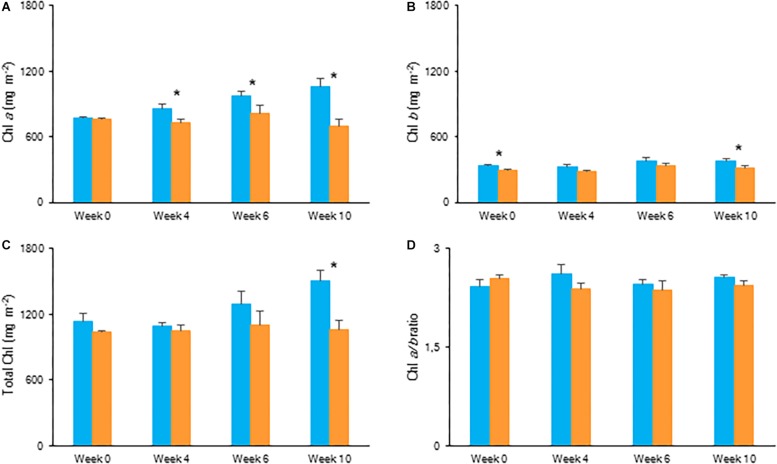
Content (mg m^–2^) of chlorophyll *a* **(A)**, chlorophyll *b* **(B)**, total chlorophyll **(C),** and chlorophyll *a/b* ratio **(D)** measured in young fully expanded leaves of *Phalaenopsis* “Edessa” at 0, 4, 6, and 10 weeks after growing under different light conditions, i.e., PPFD of 50 μmol m^–2^ s^–1^ (blue) and PPFD of 200 μmol m^–2^ s^–1^ (orange). Data are means ± SD (*n* = 5 plants). Asteriks indicate significant differences between different light conditions at *P* < 0.01 according to the independent sample *t*-test.

After 10 weeks of growing under different light conditions, significantly lower values in content of chlorophyll *a*, chlorophyll *b* and total chlorophyll were observed for plants grown under the highest light condition (Figure 6). The Chl *a*/*b* ratio remained unaffected by light condition. Both fresh and dry mass of the shoots showed an overall similar plant growth for plants grown under LL and HL after 10 weeks (Table 2).

**TABLE 2:** Biomass on fresh and dry mass (g) basis for above ground material of *Phalaenopsis* “Edessa” grown for 10 weeks under different light conditions, i.e., PPFD 50 μmol m^–2^ s^–1^ or 200 μmol m^–2^ s^–1^.

	**PPFD (μmol m^–2^ s^–1^)**	
	
	**50**	**200**
Fresh	94 ± 13	74 ± 5
Dry	4.4 ± 0.6	4.0 ± 0.4

*Data are means ± SD (*n* = 5 plants) and no significant differences between the different light conditions were detected at *P* < 0.01 according to the independent sample *t*-test.*

### Drought

#### Gas Exchange Measurements and Titratable Acidity

**FIGURE 7:**
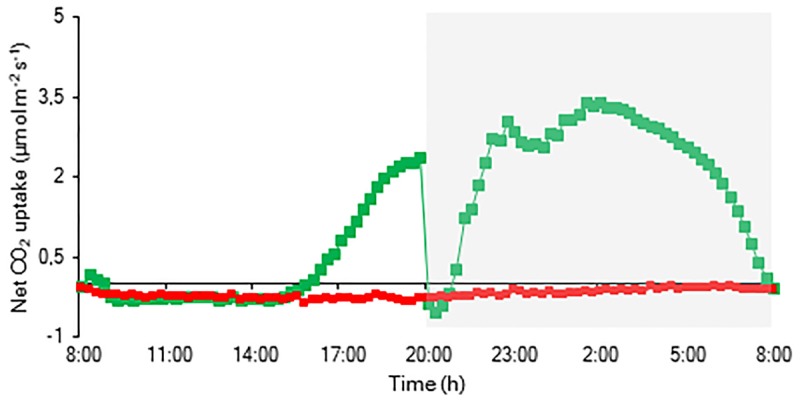
Net 24 h CO_2_ uptake (μmol m^–2^ s^–1^) for young fully expanded leaves of *Phalaenopsis* “Edessa” 6 weeks after drought stress (red) compared with the controls (green). The dark period is indicated in gray. Gas exchange curves are representative of three replicate runs with *SE* < 15%.

**FIGURE 8:**
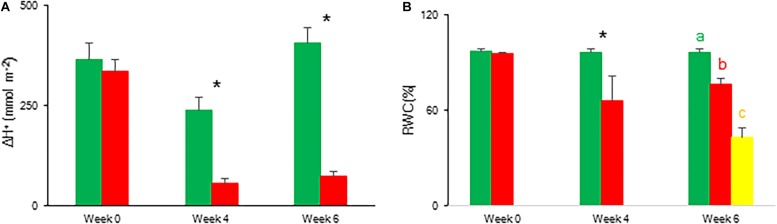
Nocturnal increase (between 20.00 h and 08.00 h) in titratable acidity (mmol H^+^ m^–2^) **(A)** and relative water content (%) **(B)** measured at 08.00 h in young fully expanded leaves of *Phalaenopsis* “Edessa” after 0, 4, and 6 weeks for controls (green) or drought stressed plants (red). At week 6 additional measurements were also made for the oldest, most wilted leaves (yellow) for relative water content. Data are means ± SD (*n* = 5 plants). Asteriks indicate significant differences between control and drought at *P* < 0.01 according to the independent sample *t*-test. At week 6 values were compared between control, drought and wilted according to Tukey’s Studentized range test at *P* < 0.01 marked by different letters.

The CO_2_ uptake pattern of *Phalaenopsis* “Edessa” grown under standard conditions (PPFD 100 μmol m^–2^ s^–1^; 12-h photoperiod), watered twice a week, fitted the four-phase CAM framework as earlier described (Figure 7). After 6 weeks of withholding water no net CO_2_ uptake occurred, indicating that the plants were in the CAM-idling mode with closed stomata during the complete diel cycle. In accordance with the gas exchange data, the nocturnal increase in titratable acidity was reduced by >80% when grown under drought stress (Figure 8A).

#### Chlorophyll *a* Fluorescence Parameters and Relative Water Content

**FIGURE 9:**
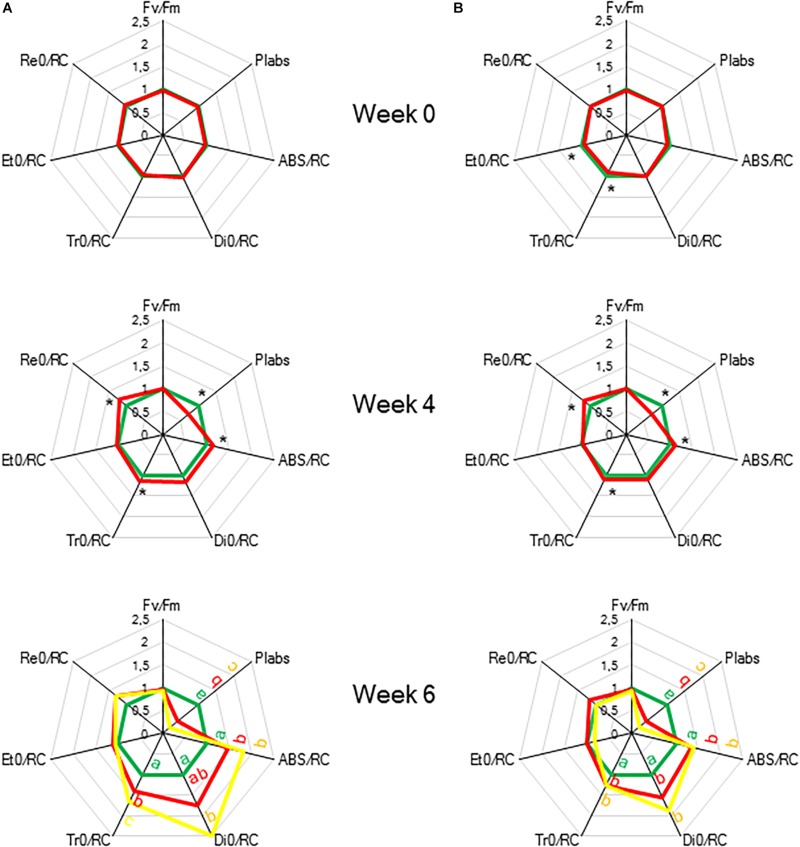
“Spider plots” of selected chlorophyll *a* fluorescence transients parameters measured at 08.00 h in young fully expanded leaves of *Phalaenopsis* “Edessa” after 0, 4, and 6 weeks for controls (green) or drought stressed plants (red). At week 6 additional measurements were also made for the oldest, most wilted leaves (yellow). Parameters are calculated without connectivity **(A)** and corrected for connectivity between PSII units **(B)**. All values are shown as percent of control. Asteriks indicate significant differences between control and drought at *P* < 0.01 according to the independent sample *t*-test. At week 6 values were compared between control, drought and wilted according to Tukey’s Studentized range test at *P* < 0.01 marked by different letters.

To indicate the onset of water stress, RWC was measured and showed a significant decrease from 4 weeks of withholding water supply (Figure 8B). During progression of drought the maximal quantum efficiency of PSII photochemistry (F_v_/F_m_) remained unaffected (Figure 9 and Table 1). In contrast, the PI_abs_ parameter was already significantly lower after 4 weeks of drought stress in comparison to control plants (Figure 9). Drought stress also affected the specific energy fluxes within and related to PSII (Figure 1A and Table 1). After 4 weeks of drought stress, a significant increase was observed for photon absorption (ABS/RC) compared to control plants (Figure 9A). Also for the trapping efficiency (Tr_0_/RC) and the flow of electrons further than PSII (Re_0_/RC), significantly higher values were obtained at week 4 in the leaves of plants exposed to drought stress (Figure 9A). At week ten Tr_0_/RC was significantly higher in the most wilted leaves whilst Re_0_/RC and Et_0_/RC remained unaffected. Activity of energy dissipation (Di_0_/RC) showed a significant increase in the most wilted leaves compared to the measurements in plants grown under control conditions.

**FIGURE 10:**
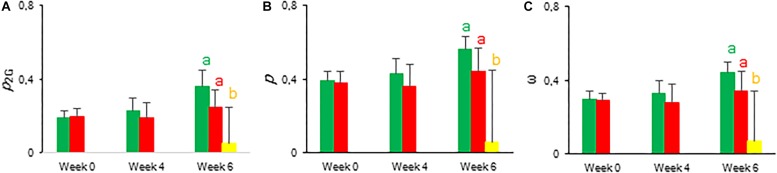
Calculated connectivity parameters derived from fast fluorescence kinetics measured at 08.00 h in young fully expanded leaves of *Phalaenopsis* “Edessa” after 0, 4, and 6 weeks for controls (green) or drought stressed plants (red). At week 6 additional measurements were also made for the oldest, most wilted leaves (yellow). **(A)** Overall grouping probability of PSII units when they are all open. **(B)** Connectivity parameter. **(C)** Probability of connectivity among PSII units when they are all closed (Strasser and Stirbet, 2001; Stirbet and Govindjee, 2011; Stirbet, 2013). Data are means ± SD (*n* = 15 plants). No significant differences were detected between control and drought at *P* < 0.01 according to the independent sample t-test. At week 6 values were compared between control, drought and wilted according to Tukey’s Studentized range test at *P* < 0.01 marked by different letters.

The calculated connectivity parameters (Table 1) of measurements performed at week 6 in leaves of control plants were ∼1.3 times higher compared to measurements performed on the youngest fully expanded leaves of plants exposed to drought stress, and ∼7.6 times higher compared to measurements on the most wilted leaves of plants exposed to drought stress (Figure 10). As a consequence, the increase of the calculated fluxes after correction for connectivity, was the highest in the control leaves and the lowest in the most wilted leaves (Figure 9B). Taking connectivity into account showed an increased energy dissipation (Di_0_/RC) for drought stressed plants and indicated an equal trapping efficiency (Tr_0_/RC) for drought and wilted leaves at week six (Figure 9B).

#### Chlorophyll Content

**FIGURE 11:**
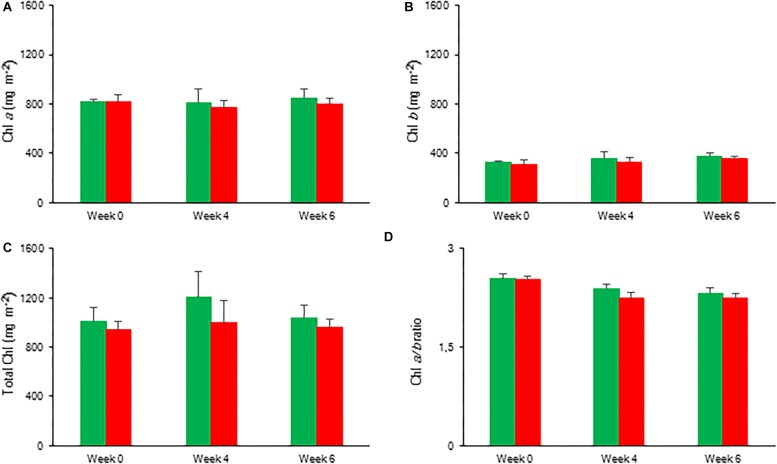
Content (mg m^–2^) of chlorophyll *a* **(A)**, chlorophyll *b* **(B)**, total chlorophyll **(C)**, and chlorophyll *a/b* ratio **(D)** measured in young fully expanded leaves of *Phalaenopsis* “Edessa” after 0, 4, and 6 weeks for controls (green) or drought stressed plants (red). Data are means ± SD (*n* = 5 plants). No significant differences were detected between control and drought at *P* < 0.01 according to the independent sample *t*-test.

Overall, the content of photosynthetic pigments remained unaffected during the 6 weeks of drought stress. No significant differences were observed for Chl *a*, Chl *b*, and total Chl between control and drought stressed plants (Figure 11).

## Discussion

Chlorophyll fluorescence measurements in CAM plants mainly report photochemical and non-photochemical quenching (qP and qN), the maximum quantum efficiency (F_v_/F_m_) and the effective quantum yield (*Φ*_PSII_) of PSII photochemistry (Table 1; Winter and Demmig, 1987; Winter et al., 1990; Keiller et al., 1994; Adams and Demmig-Adams, 1996; Heber et al., 1996; Barker and Adams, 1997; Skillman and Winter, 1997; de Mattos et al., 1999; Lin and Hsu, 2004; Masrahi et al., 2015; Liu et al., 2016; Arias-Moreno et al., 2017). A detailed investigation of the performance index, specific energy fluxes related to PSII processing and the concept of connectivity between PSII units (Figure 1 and Table 1) revealed important PSII related adaptations concerning the initial light reactions in CAM plants under different environmental conditions.

### Increased PSII Connectivity Under Light Limitation

Chlorophyll fluorescence measurements were performed to investigate the effects of low light (LL: PPFD 50 μmol m^–2^ s^–1^) and high light (HL: PPFD 200 μmol m^–2^ s^–1^) on the photosynthetic performance of the CAM plant *Phalaenopsis* “Edessa.” Our detailed energy flux analyses focused on the efficiency of energy capture and the electron transport in PSII system when all RCs are open (i.e., at the onset of excitation). Comparing these parameters between LL and HL (Figure 4), stresses the importance of the energetic communication between PSII complexes defined as connectivity when evaluating specific energy fluxes in CAM plants (Figure 1). Without taking connectivity into account (Figure 4A), all values of specific energy fluxes were significantly lower after 10 weeks of growing under LL compared to the HL treatment, indicating an affected energy processing through PSII upon light limitation. However, taking into account higher connectivity of LL grown plants (Figure 5), higher values were recorded for all energy fluxes (Figure 4B) and as such these plants seemed to process light energy more efficiently compared to plants grown under the high light conditions. This was also reflected in a 25% higher maximum quantum yield for plants under LL as indicated by relating the daily CO_2_ gain of a leaf to the amount of photons absorbed per day (Skillman, 2008). Plants grown under LL realized an overall similar biomass on both fresh and dry mass basis (Table 2) despite a triple decrease in CO_2_ fixation in the measured source leaves for plants under LL (Figure 2). However, HL grown plants were typically characterized by several layers of leaf pairs largely shading each other whilst LL grown plants showed an improved architecture with less underlying sink leaves and larger source leaves. Under LL, total chlorophyll content in source leaves after 10 weeks was also 140% in comparison to contents of plants grown under HL (Figure 6C), which was also reflected by the higher photon absorption flux (ABS/RC) for plants grown under LL (Figure 4B). The increase in total chlorophyll content was mainly due to a significant increase in Chl *a* (Figure 6A), which can reflect an increase in active RC’s. As a consequence, the higher connectivity in plants grown under LL is consistent with the absence of a difference in the performance index PI_abs_ (Figure 4 and Table 1), due to their more efficient energy processing. Without considering connectivity an increase in PI_abs_ would be expected for HL, since both trapping efficiency and electron transport increased significantly by HL and only the parameter RC/ABS decreased. Taking connectivity into account changed the concentration of reaction centres per chlorophyll (RC/ABS) and trapping efficiency (Tr_0_/RC) in the opposite direction, whilst the other parameters remained unaffected and no difference was observed for PI_abs_. As such, the question can be raised whether a higher connectivity for plants grown under low light conditions might underpin the adaptive radiation of CAM plants from full sun to deep shade conditions. Several species from the Bromeliaceae are excellent examples of CAM representatives thriving in light limited environments such as wet cloud forests and shaded understoreys of tropical rainforests (Pierce et al., 2002; Ceusters et al., 2011).

### Increased Energy Dissipation and Partial Inactivation of PSII Reaction Centres to Reconcile Energy and Carbon Requirements During Drought Stress

To evaluate the effects of drought stress on the photosynthetic performance in the CAM plant *Phalaenopsis* “Edessa,” chlorophyll fluorescence parameters (Table 1) were compared between control plants (watered twice a week) and drought stressed plants. In contrast to the maximum quantum efficiency of PSII (F_v_/F_m_) which remained unaffected, the performance index PI_abs_ reflected the reduction of RWC for drought stressed plants (Figure 9). These results corroborate investigations in C3 plants where the performance index PI_abs_ has already been proposed a reliable and sensitive parameter to evaluate plant homeostasis with regard to moderate abiotic stress (Zivcak et al., 2008; Campos et al., 2014; Jedmowski et al., 2015). As indicated in Table 1, the performance index takes into account three dependent characteristics: the concentration of reaction centres per chlorophyll (RC/ABS), a parameter related to primary photochemistry (φP_0_) and a parameter related to electron transport (ψE_0_) (Strasser et al., 2004). As a consequence, changes in environmental conditions which influence either antenna properties (ABS/RC), trapping efficiency (Tr_0_/RC) or electron transport beyond Q_A_ in PSII (Et_0_/RC and Re_0_/RC) (Figure 1) will have an impact on the current state of plant performance and PI_abs_ will decrease under stress. Mathematically, the observed decrease in performance index could not be explained by merely looking at the three separate parameters on which PI_abs_ is based (Table 1). Only the trapping efficiency (Tr_0_/RC) significantly increased under drought whilst RC/ABS was only significantly different between control and drought, and the parameter Et_0_/RC remained unaffected (Figure 9A). Taking connectivity into account eliminated the observed significant difference for Tr_0_/RC between drought and wilted but could not fully explain the observed significant decrease in PI_abs_ (Figure 9B). From these data it can be concluded that other components or processes, such as oxygen-evolving complex (OEC); transition from active to non-Q_A_-reducing centres; carotenoids and PSI may also influence the performance index (Figure 1). Different studies revealed that drought can indeed result in damage to the OEC and block further electron transport. This phenomenon is mostly characterized by an increase in the specific fluxes for trapping, absorption and dissipation (Guissé et al., 1995; Wang et al., 2012; Guha et al., 2013).

Our data also demonstrate that enhanced protection of photochemistry in the CAM plant *Phalaenopsis* “Edessa” is also achieved by adjusting the energy distribution between photosystems. Two possible explanations can be presented for the observed increase in ABS/RC under drought stress (Figure 9B): (1) inactivation of some PSII RC’s, considering that the ABS/RC ratio is calculated as the total number of photons absorbed by Chl molecules in all RC’s divided by total number of active RC’s, or (2) an increase in antenna size (van Heerden et al., 2007; Gomes et al., 2012). The latter seems to be less plausible since no significant differences were obtained in the content of photosynthetic pigments between the two treatments (Figure 11). The inactivation of some PSII RC’s, becoming non-Q_A_-reducing centres, can explain the observed increase in Tr_0_/RC under drought. Down-regulation of photochemical activity together with a significant increase in thermal dissipation may play a critical role in protecting plants exposed to drought stress from over-excitation and photodamage (van Heerden et al., 2007; Zivcak et al., 2014b; Wang et al., 2017; Kalaji et al., 2018). As such, the performance index depicts a downregulation of PSII related energy fluxes as a physiological response to drought stress. Especially for CAM plants during CAM idling the Calvin-Benson cycle, for which the end products of the electron transport chain (ATP and NADH) are indispensable (Figure 1A), is seriously compromised by carbon and water limitation (Figure 7). In this physiological mode of survival, inactivation of several PSII RC’s adds in overcoming severe drought periods by attenuating energy fluxes in consistence with the very low CO_2_ availability, due to stomatal closure during day and night, in the leaf mesophyll cells.

## Conclusion

The results presented in this paper suggest a clear physiological role for PSII connectivity (energetic communication between PSII complexes) in the CAM plant *Phalaenopsis* “Edessa.” A higher connectivity for plants grown under low light (*p* ∼ 0.51) compared to plants grown under high light (*p* ∼ 0.31) resulted in similar specific energy fluxes irrespective of the light treatment. With a 25% higher maximum quantum yield and comparable biomass formation, low light grown plants proved to process light energy more efficiently compared to high light grown plants. As such, taking into account PSII connectivity might help to better understand the adaptation of CAM plants to changing environmental conditions. The obtained results also indicate that PI_abs_ is a sensitive parameter to detect drought stress in the CAM plant *Phalaenopsis* “Edessa.” Besides an enhanced thermal dissipation drought stressed plants also seemed to attenuate the electron flow from PSII toward PSI by partial inactivation of PSII reaction centres. This strategy might help to reconcile the light reactions and the carbon fixation reactions which are seriously compromised by carbon and water limitation during CAM-idling and as such minimize chances of photodamage to occur. Further research into the specific energy fluxes and the connectivity between different PSII units in CAM plants under combinations of abiotic stress factors is highly encouraged. This will enable to expand our physiological knowledge about the interplay of the light and carbon fixation reactions in CAM plants under changing environmental conditions.

## Data Availability

The raw data supporting the conclusions of this manuscript will be made available by the authors, without undue reservation, to any qualified researcher.

## Author Contributions

NC, RV, and JC proposed the conceptual framework for the study and performed the data collection and analysis. NC and MF performed the experimental analyses. NC, RV, JEC, WVdE, and JC interpreted the data and wrote the manuscript.

## Conflict of Interest Statement

The authors declare that the research was conducted in the absence of any commercial or financial relationships that could be construed as a potential conflict of interest.
