# Supplementary material for: Performance Index and PSII Connectivity Under Drought and Contrasting Light Regimes in the CAM Orchid Phalaenopsis
Source: Front Plant Sci. 2019 Aug 6;10:1012. doi: 10.3389/fpls.2019.01012 (PMC6691161; doi:10.3389/fpls.2019.01012)
Supplement: Supplementary file 1 [file Table_1.docx]

Supplementary Material

**Supplementary Table 1.** Changes in chlorophyll *a* fluorescence transients measured at Phase II and III in young fully expanded leaves of *Phalaenopsis* ‘Edessa’ ten weeks after growing under different light conditions (PPFD)

|  | PPFD (µmol m^-2^ s^-1^) | | |
| --- | --- | --- | --- |
|  |  | 50 | 200 |
|  |  |  |  |
| ABS/RC | Phase II | 2.12 ± 0.42 | 2.37 ± 0.33 |
|  | Phase III | 1.84 ± 0.61 | 2.47 ± 0.31 |
|  |  |  |  |
| Tr_0_/RC | Phase II | 1.74 ± 0.35 | 1.94 ± 0.28 |
|  | Phase III | 1.50 ± 0.50 | 2.01 ± 0.27 |
|  |  |  |  |
| Et_0_/RC | Phase II | 1.12 ± 0.26 | 1.11 ± 0.08 |
|  | Phase III | 1.06 ± 0.35 | 1.22 ± 0.15 |
|  |  |  |  |
| Re_0_/RC | Phase II | 0.27 ± 0.05 | 0.29 ± 0.04 |
|  | Phase III | 0.33 ± 0.11 | 0.33 ± 0.06 |
|  |  |  |  |
| Di_0_/RC | Phase II | 0.39 ± 0.07 | 0.43 ± 0.06 |
|  | Phase III | 0.34 ± 0.11 | 0.45 ± 0.05 |
|  |  |  |  |

Data are means ± SD (n = 15) and no significant differences were detected between Phase II and Phase III at P < 0.01 according to the independent sample t-test
